# Screening of Tomato Seed Bacterial Endophytes for Antifungal Activity Reveals Lipopeptide Producing *Bacillus siamensis* Strain NKIT9 as a Potential Bio-Control Agent

**DOI:** 10.3389/fmicb.2021.609482

**Published:** 2021-06-10

**Authors:** Ayushi Sharma, Nutan Kaushik, Abhishek Sharma, Abhay Bajaj, Mandar Rasane, Yogesh S. Shouche, Takwa Marzouk, Naceur Djébali

**Affiliations:** ^1^Amity Food and Agriculture Foundation, Amity University Uttar Pradesh, Noida, India; ^2^Amity Institute of Microbial Technology, Amity University Uttar Pradesh, Noida, India; ^3^National Centre for Microbial Resource, National Centre for Cell Science, Pune, India; ^4^Centre of Biotechnology of Borj Cedria, Laboratory of Bioactive Substances, Hammam-lif, Tunisia

**Keywords:** crop protection, bio-pesticide, diversity indices, plant growth promotion, UPLC, antagonistic

## Abstract

The current study investigates the diversity pattern and fungicidal potential of bacterial endophytes isolated from two different organic varieties of tomato plants (V1 and V2). A total of seventy-five bacterial isolates identified by 16S rRNA gene sequencing revealed a majority of genus as *Bacillus* and one *Planococcus*, which were grouped into eight different species. The Shannon diversity H’ (1.56), Simpson’s index of diversity (0.93), Magalef’ index (2.23), Evenness (0.96), and Species richness (7) indicated the high endophytic bacterial diversity in the V1 variety of the tomato. Bacterial endophytes isolated from both of the varieties were screened for their antifungal activity against five economically critical fungal pathogens (viz., *Botrytis cinerea*, *Rhizoctonia solani*, *Fusarium solani*, *Verticillium lateritium*, and *Alternaria solani*) of tomato crop through dual culture assay. The data revealed *B. siamensis* strain NKIT9 as the most potent antagonist, significantly (*p* < 0.05) inhibiting the mycelial growth between 75 to 90% against selected fungal pathogens. High bioactivity of lipopeptide extract of strain NKIT9 was recorded against *R. solani* with minimum IC_50_ value of 230 μg/ml. The Ultra Performance Liquid Chromatography-High Definition Mass Spectrometry (UPLC-HDMS) analysis of this lipopeptide extract revealed the presence of Surfactin and Bacillomycin D. Furthermore, *in-vitro* results showed that the selected bacterial strain significantly minimized the disease incidence in damping-off assay which makes this strain a promising antifungal bio-control agent. Moreover, in the pot experiment the NKIT9 increased the fruit yield by 59.2% compared with the untreated *R. solani* infested control.

## Introduction

Tomato (*Solanum lycopersicum*) is a well-known vegetable crop due to its high nutritional values, which suffers from various fungal infestation diseases. Its succulent fruit increases its susceptibility toward fungal attacks compared to other crop plants, which is a critical limiting factor in its production (Habiba et al., 2017). The major phytopathogens responsible for damaging this crop include *Rhizoctonia solani*, *Fusarium solani, Botrytis cinerea, Alternaria solani*, and *Verticillium sp.* because of their diverse host spectra and soil-borne existence, fungal phytopathogens are difficult to control (Lamichhane et al., 2017). *R. solani* is one of the most important and widespread soil-borne phytopathogenic fungi causing yield losses in more than 200 crops around the world (Sturrock et al., 2015) via seedling damping-off and crown rot (Montealegre et al., 2010; Zamoum et al., 2017). *A. solani* can cause serious damage during all stages of plant growth (Attia et al., 2020). It has been reported to damage photosynthesis and pigment content, resulting in substantial growth inhibition and thus a major reduction in yield (Agamy et al., 2013). Gray mold, caused by *B. cinerea*, is a significant and persistent threat to tomatoes grown in fields and greenhouses in many countries around the world (Soylu et al., 2010). Site-specific fungicides such as benzimidazoles, dicarboximides, and Nphenylcarbamate were predominantly used to control gray fungus (Leroux et al., 2002). While synthetic fungicides are successful, their prolonged or persistent use has weakened biological control by natural enemies, resulting in disease outbreaks and widespread production of fungicide resistance (Soylu et al., 2010).

Soil-borne diseases caused by fungi such as *F. solani* are often a significant crop production constraint. *F. solani* is responsible for root rot in tomato plants (Shenashen et al., 2017). Chitin-containing pathogens including *F. solani* and *A. solani* cause the most damage to crop plants among fungal pathogens and can become chemical fungicide resistant (Badrhadad et al., 2018). *Verticillium*, a soil-borne fungus, causes serious vascular disease in a wide range of vegetable crops. In dead or dying plant tissues, *Verticillium* species develop long-lasting settling structures such as microsclerotia, chlamydospores, and resting mycelium (Deketelaere et al., 2017). Such resting complexes act as the primary inoculum, forming hyphae that penetrate the roots of host plants directly (Deketelaere et al., 2017). The use of chemical fungicides is the most common strategy to prevent fungal pathogens (Windels and Brantner, 2005). The prolonged use of chemicals has led to the development of resistance in phytopathogens, such as Fluazinam, fentin chloride, fludioxonil, difenoconazole, 28 cyazofamid, chlorothalonil, and 2, 4-dinitrophen, which have all been reported to cause multi drug resistance in *R. solani* (Cheng et al., 2020).

Seeds are the key structures of plants that help them to not only reproduce over time, but also to survive stress in the most effective way possible; as a result, seeds play an important role in agriculture (Guan et al., 2009). Seeds harbor bacterial endophytes (López et al., 2018) which play an essential task in managing plant health and diseases (Hazarika et al., 2019) by reducing pathogen population densities without stimulating hypersensitive reactions in the host (Roy et al., 2017; Hazarika et al., 2019). Bacterial endophyte composition varied among plants, organs, genotypes, tissues, cultivars, soil, and location (Kumar et al., 2020). The rhizosphere or phyllosphere work as a source for several endophytes; nevertheless, some bacterial species have been reported as having vertical transmission through seeds (Truyens et al., 2014). Plants choose unique microbial communities to establish association, such as rice plants grown in neutral-pH soil supporting seed-borne *Pseudomonas oryzihabitans* and *Rhizobium radiobacter*, while plants grown in low-pH soil favor *Enterobacter*-like and *Dyella ginsengisoli* (Hardoim et al., 2012). The potential benefits of endophytic bacteria are close to those of rhizosphere bacteria (Ryan et al., 2008) including serving as biological control agents (Hong and Park, 2016). Many endophytic bacteria, mainly *Bacillus* species, exhibit antagonistic ability toward fungal pathogens. Studies have reported the antifungal ability of *Bacillus subtilis* SCB-1 against diverse fungal pathogens, including the *Alternaria* and *Fusarium* (Hazarika et al., 2019). In another study, volatile organic compounds of highly antagonistic *Bacillus* strains were reported against *Sclerotinia sclerotiorum* (Massawe et al., 2018). *Bacillus* species is becoming an attractive agent for commercial use in modern farming systems (Piggot and Hilbert, 2004; Tiago et al., 2004) due to their heat and UV resistant spore forming capacity which can withstand adverse environmental conditions.

In the present study, the diversity of the endophytic bacteria isolated from the various tissues of two different organic tomato varieties was evaluated for its bio-control potential against tomato pathogens.

## Materials and Methods

### Seed Collection

Two organic tomato varieties were used in this study for the isolation of bacterial endophytes. Both the varieties, i.e., Pusa Ruby (Maharashtra) (V1) and a local variety of Andhra Pradesh (Madanapalle) (V2) were procured from the online garden stores, Ugaoo and Organic Garten, respectively.

### Isolation of Bacterial Endophytic Strains

Surface sterilization of tomato seeds was performed following the method described by Kumar et al. (2011). Briefly, seeds were first sterilized with 70% ethanol for 2 min, followed by a 1% sodium hypochlorite solution for 3 min. After that, surface-sterilized seeds were washed three times with autoclaved distilled water and dried with sterile blotting paper. For sterility check, imprints of dry surface-sterilized seeds were taken on Luria-Bertani agar medium (10 g Peptone 140; 5 g Yeast Extract; 5 g Sodium Chloride; 12 g Agar) (Vinodkumar et al., 2018). Seeds were then put for germination on sterile filter paper immersed with autoclaved distilled water in a petri dish at 27°C. For isolation (Chimwamurombe et al., 2016), seedlings obtained after the 9 days of germination were again surface sterilized with the method described above. After the sterility check, each seedling was cut into different sections viz., root, hypocotyl, and cotyledon. Each part was further divided into various segments and placed on the Luria-Bertani agar plate. Plates were then incubated for 2–3 days at 27°C. Visually distinct bacterial colonies acquired from segmented seedlings were purified and maintained in Nutrient agar slants/plates and glycerol stock at 4 and −80°C, respectively. The identification of the most active strain among the isolates was further confirmed with endospore staining as per the method of Aneja (2003).

### Molecular Identification of Bacterial Endophytes and Phylogenetic Analysis

The identification of isolates was carried out at the Sequencing facility of the National Centre for Microbial Resource (NCMR), National Centre for Cell Science, Pune. DNA extraction and purification was done using HiPurA 96 Bacterial Genomic DNA Purification Kit (Himedia), as per the manufacturer’s protocol; followed by amplification of the 16S rRNA gene using universal bacterial primers (27F and 1492R). PCR amplification was performed with initial denaturation for 5 min at 94°C followed by 30 cycles of 30 s denaturation at 94°C, 30 s of primer annealing at 55°C, 1 min elongation at 72°C, and final extension of 7 min at 72°C. Amplified products were sequenced by the Sanger method on ABI 3730xl Genetic Analyzer (Applied BioSystems). The sequences were aligned and evaluated for taxonomic identification by BLASTn analysis (Boratyn et al., 2013). The phylogenetic tree was reconstructed by doing alignment using Muscle and the evolutionary history inferred using the neighbor-joining (NJ) method. A tree with 100 bootstrap replicates was constructed using MEGA-7 (Kumar et al., 2016).

### Diversity Indices of Bacterial Endophytes

Bacterial endophytes derived from organic tomato seedlings were grouped into their specific isolation sections, such as hypocotyl, root, and cotyledon, which facilitated the comparison between the isolates of the same or other variety. Species diversity was calculated using the Shannon diversity index to measure species evenness and richness (Chowdhary and Kaushik, 2019).

$$H^{\prime }=-\sum_{i=1}^{s}p_{i}\text{In}\left(p_{i}\right)$$

Where, *S* equals the number of species, and *pi* equals the ratio of individuals of species *i* divided by all individuals *N* of all species. The Shannon diversity index ranges typically from 1.5 to 3.5 and rarely reaches 4.5. Simpson’s index (D) was calculated to determine the dominance, the higher the value lower in the diversity (Ifo et al., 2016).

$$D=\sum_{i=1}^{s}\left(\frac{{\text{n}}_{\text{i}}\left({\text{n}}_{\text{i}}-1\right)}{\text{N}\left(\text{N}-1\right)}\right)$$

Where, n_*i*_ is the number of individuals in the i^*th*^ species and *N* equals the total number of individuals and Simpson’s index of diversity was calculated b

$$D^{\prime }=\left(1-D\right)$$

Other parameters, such as species evenness and richness, were also calculated (Ifo et al., 2016). Margalef’s index (*d*) also indicates the evenness (Kumar et al., 2006).

$$d=\frac{\left(S-1\right)}{In\left(N\right)}$$

*S* is the total number of species; *N* is the number of individuals, and the natural logarithm.

To measure the similarity in the species composition for both varieties of tomato, we used Sorenson’s index of similarity using the equation,

QS= 2a/(2a+b+C)

and, Jaccard’s index of similarity using the equation,

JS=a/(a+b+C)

Whereas, “*a*” denotes the number of bacterial species commonly shared by both the varieties, “*b*” denotes the number of bacterial species found in V1, and “*c*” denotes the number of bacterial species found in V2 (Chowdhary and Kaushik, 2015).

### *In-vitro* Antifungal Activity of Bacterial Endophytes

All the bacterial isolates were screened for their antagonistic activity against major pathogenic fungi of the tomato crop, namely, *R. solani* (ITCC-6430), *F. solani* (ITCC-6731), *B. cinerea* (ITCC-6011), *A. solani* (ITCC-4632), and *V. lateritium* (ITCC-2819) obtained from Indian Type Culture Collection (ITCC) at Indian Agricultural Research Institute (IARI), Pusa, New Delhi, India. Isolates were evaluated by dual culture assay on Potato Dextrose Agar (PDA) medium (Slama et al., 2019). A fully grown 7 mm fungal disc was placed in the center of the PDA plate in an inverted position to aid the contact of the fungal mycelium with the culture medium, while bacterial isolate was streaked on both sides of the fungal disc at equidistance. A PDA plate inoculated only with the fungal disc was kept as the control. After 5–7 days of incubation, plates were observed for the antagonism expressed by endophytic bacteria, and percentage growth inhibition was calculated. Experiment was performed in the replication of three. Growth inhibition (GI) was calculated as per the following:

GI={(A-B)/A}×100

Where, *A* = radial growth of the plant pathogenic fungus in control; *B* = radial growth of the plant pathogenic fungus in the presence of endophytic bacterial strain (dual inoculation). Amongst all the isolates, only *B. siamensis* strain NKIT9 showed the highest antifungal activity against all test pathogens. Therefore, the *B. siamensis* strain NKIT9 was selected for further studies.

### Extraction and Identification of Lipopeptide

The endophytic bacteria *B. siamensis* strain NKIT9, with the most promising antagonistic activity against all the test pathogenic fungi, was further explored for the production of antifungal lipopeptides. The lipopeptide extraction method involved acid precipitation and solvent extraction, as described by Romano et al. (2011). Briefly, extraction of lipopeptide from a cell-free supernatant was done by precipitation method at pH 2 using 6N HCl and incubated at 4°C overnight and then centrifuged at 12,000 rpm for 15 min at 4°C. The pellet was dissolved in a solvent mixture of Chloroform: Methanol (2:1, v/v) followed by centrifugation for at 12000 rpm for 15 min at 4°C to remove any undissolved extract. The lipopeptides present in the supernatant were filtered and concentrated to dryness by rotary evaporation. Waters ACQUITY Ultra Performance Liquid Chromatography (UPLC) H-class system with the BEH C18 column was employed for lipopeptide profiling. The lipopeptide extract (10 mg) was dissolved in 10 mL of HPLC grade ethanol and filtered through 0.22 μm PVDF membrane syringe filter (Durapore, Merk), and a 10 μL aliquot of the sample was injected into the column and the column was eluted at a flow rate of 0.3 ml/min with 100% methanol at 0 min and gradually increased the polarity to acetonitrile: methanol (40:60) for 4 min which was further retained for 22 min followed by 100% methanol for 25 min. The lipopeptides were detected by mass spectroscopy using positive ionization electrospray (ESI+) Synapt G2-Si High Definition Mass Spectrometer (HDMS) coupled with the UPLC system.

### Antifungal Bioassay of the Lipopeptides

The lipopeptide extracted from cell free supernatant of bacterial *B. siamensis* strain NKIT9 was tested against all five phytopathogenic fungi viz. *R. solani, F. solani, B. cinerea, A. solani*, and *V. lateritium*, by poisoned food technique (Ibrahim et al., 2017). From a 40 mg/ml stock solution prepared in methanol, the extract was added to autoclaved molten PDA at the final extract concentration of 50, 100, 250, 500, and 1000 μg/ml to prepare the agar plates. The fungal pathogens were inoculated by placing a 7 mm agar disc at the center of every petri plate in an inverted position for the greater contact of fungal mycelium and were incubated at 27°C for 5 days under dark conditions. Eight replicates were used for each concentration. In parallel, PDA mixed with 250 μl of methanol inoculated with fungal disc served as the control. Percentage of growth inhibition (% GI) was calculated by comparing the radial distance of fungal growth toward each spot inoculation with ethanol control. IC_50_ was calculated using regression equation analysis.

### *In vivo* Bio-Control Assay

#### Bio-Priming of Tomato Seeds

Tomato seeds (Pusa Ruby) were surface sterilized by the above described method. Surface sterilized seeds were bio-primed with pure culture of *B. siamensis* strain NKIT9, with the microbial load adjusted to ≥10^8^ cfu/ml by diluting with sterile saline water. In contrast, uncoated surface-sterilized seeds were kept as the control. Seeds were then kept for incubation with continuous agitation (150–200 rpm) at 27°C for 24 h (Xia et al., 2015). Bio-primed seeds were placed on sterile blotting paper and air dried in laminar air-flow.

#### Damping-Off Assay

Based on the in vitro antifungal activities, strain NKIT9 was selected for *in vivo* assay for bio-control of damping-off of tomato seedlings caused by *R. solani*. Experiment was carried out in germination trays (Hyco trays) with ten cavities in each. Six treatments were performed:

T1: Surface-sterilized seeds only (control),

T2: Surface-sterilized seeds + fungal pathogen (*R. solani*),

T3: Bio-primed seeds only (for growth promoting activity),

T4: Bio-primed seeds + fungal pathogen,

T5: Bio-primed seeds + fungal pathogen + soil treatment of the bacterial strain NKIT9,

T6: Chemical treated seeds (Bavistin-Carbendazim) + fungal pathogen (chemical control);

There were nine replicates for each treatment with 10 seedlings in each replicate. In the chemical control, sterilized seeds were treated with Carbendazin 50% WP as recommended by the manufacturer (2 g/kg of sterilized seeds) for 1 h before sowing (Adhikari et al., 2015). For each treatment, one tomato seed was sown per cavity of the tray as per the treatments. Each tray was filled with an autoclaved mix of cocopeat: vermiculite: perlite (2:1:1). The pathogen inoculation was done with sorghum seeds colonized by *R. solani* with a spore density of 10^4^ cfu/g (Khan and Anwer, 2007; Paul, 2018). For the application of strain NKIT9 in the soil, 72 h old bacterial culture pellets were washed and re-suspended in distilled water and applied to the tray mix (10^9^ CFU/ml) (Szczech and Shoda, 2006). Trays containing tomato seeds were kept under standard conditions in a plant growth room with 23–27°C temperature and 12 h of photo period (Goudjal et al., 2014) and moisture was maintained by spraying tap water as per the requirement for seed germination. After 15 days of germination, germination and disease percentage were calculated, shoot length was measured, and seedling fresh weight was estimated.

#### Pot Experiment

Pot experiment of the tomato seeds (Pusa Ruby) was carried out in a greenhouse condition with temperatures ranging from 23–30°C and relative humidity from 60–85%, in a Completely Randomized Design (CRD). Six treatments were conducted in pot trial as described above (T1–T6) with 16 replicates for each treatment. However, in this case soil treatment with the pathogen in treatment T2 and T4 and the soil treatment of the bio-control agent in treatment T5 were given in the pot after the transplantation and no treatment was given at the nursery stage except bio-priming of the seed.

Seeds (bio-primed/non-primed seeds) were sown in hyco trays containing an autoclaved mix of cocopeat: vermiculite: perlite (2:1:1). Trays containing tomato seeds were kept under standard conditions in a plant growth room with a maintained temperature of 23–27°C and 12 h of photo period as mentioned above. Trays were watered with spraying tap water as per the requirement to maintain the moisture level favorable for seeds and the seedling. The seedlings were maintained in the trays until the four-leaf stage and then transplanted in the pots as described below.

Sandy loam soil was used for the pot study. Pot mix comprising soil and farm yard manure (2:1) was autoclaved in polypropylene plastic bags. The pots (10 inches) were filled with the pot mix and watered before sowing. For pathogen infested soil, *R. solani* inoculated sorghum seeds were mixed with the pot mix (3 g/kg) (Asaka and Shoda, 1996). Pots were then watered with sterile distilled water and incubated at room temperature for 5 days to enhance the growth of the pathogen. Plate count method was used to evaluate the density of *R. solani* in the infested soil (10^4^ CFU/g). Seedlings from the hyco trays as mentioned above were transplanted into the pots, and each pot contained one seedling. Plants were observed for number of leaves, plant height, number of flowers, and fruit yield for 70 days (from the date of sowing).

### Data Analysis

The aligned 16S rRNA gene sequences were curated and trimmed to infer the evolutionary history with 100 bootstrap replicates in the NJ method. Heatmap was produced through online software Heatmapper^1^. The data of in planta assays were analyzed by IBM SPSS Statistics 20. One-way ANOVA analysis of variance was performed for damping-off bio-control assay and pot experiment. To analyze the significant difference among the groups was determined by Post hoc different examination test (Duncan’s Multiple Range test).

## Results

### Isolation, Identification, and Phylogenetic Analysis

**FIGURE 1:**
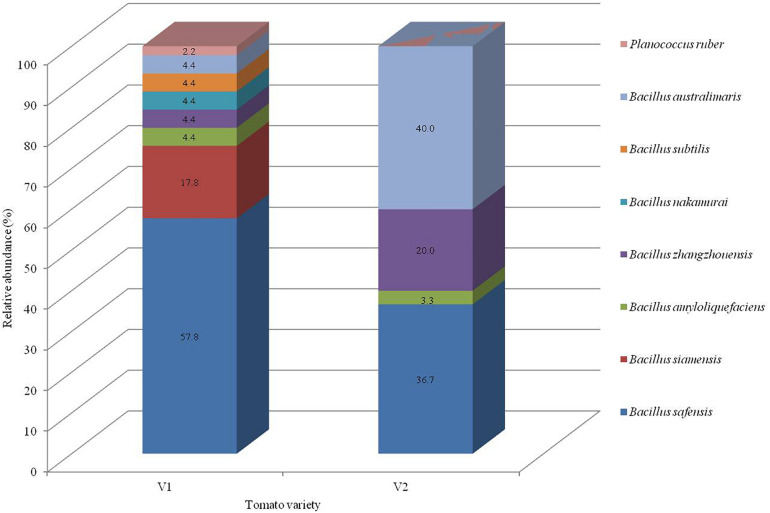
Taxonomic profiles of the bacterial community in each variety at the representative species level with the relative abundance.

**FIGURE 2:**
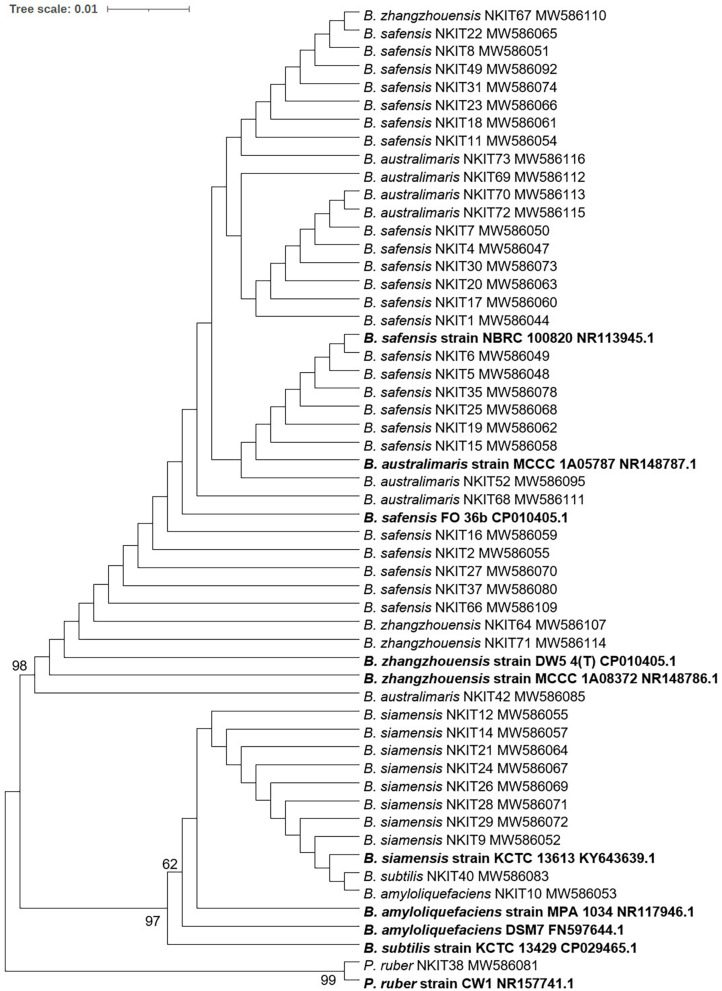
Evolutionary relationships of bacterial isolates investigated in this study: Phylogenetic tree was constructed using 16S rRNA gene sequences and the bootstrap values (100 replicates) are indicated (BS > 50) at the nodes. Closest neighbor of strain NKIT38, *Planococcus ruber* strain CW1 was used outgroup. The evolutionary history was inferred using the NJ method. The optimal tree with the sum of branch length = 0.07256030 is shown. The evolutionary distances were computed using the Maximum Composite Likelihood method and are in the units of the number of base substitutions per site. The analysis involved 55 nucleotide sequences. All positions containing gaps and missing data were eliminated. There were a total of 545 positions in the final dataset. Evolutionary analyses were conducted in MEGA7.

Seventy five bacterial endophytes were isolated from the various tissues of root, hypocotyl, and cotyledon of tomato plants of both the organic varieties (V1 and V2) using the culture-dependent technique. The majority of the isolates (60%) were obtained from the V1 variety. All the 75 isolates were grouped into eight species on the basis of 16S rRNA gene sequencing based molecular identification. Comparing the two varieties, Pusa ruby (V1) harbored all the eight species identified while the local variety (V2) possessed less diverse endophytic populations as only four species inhabited it. All the bacterial isolates belonged to the phylum Firmicutes. The details of isolates according to their closest and representative species concerning identification, accession number, similarity percentage, and source are summarized in Supplementary Table 1. In the V1 variety, *Bacillus safensis* (strains NKIT1, NKIT2, NKIT4-8, NKIT11, NKIT15-20, NKIT22-23, NKIT25, NKIT27, NKIT30, NKIT31, NKIT34, NKIT35, NKIT37, NKIT41, NKIT43, and NKIT45) and *B. siamensis* (strains NKIT9, NKIT12, NKIT14, NKIT21, NKIT24, NKIT26, NKIT28, and NKIT29) were the dominant species with relative abundance (RA) of 57.8 and 17.8%, respectively. The majority of isolates belong to the genus *Bacillus* except for one non-bacillus species, which was isolated from V1 and was identified as a member of the genus *Planococcus* (NKIT38). In V2, *Bacillus australimaris* (strains NKIT51, NKIT52, NKIT55, NKIT58, NKIT59, NKIT62, NKIT68-70, NKIT72, NKIT73, and NKIT75) and *B. safensis* (strains NKIT46, NKIT48-50, NKIT53, NKIT54, NKIT57, NKIT60, NKIT65, NKIT66, and NKIT74) were the dominant species with RA of 40 and 36.7%, respectively (Figure 1). In V1 isolates, only two endophytic bacterial species, namely *B. safensis* and *B. siamensis*, were isolated from all the three parts of tomato seedling such as root (strains NKIT4, NKIT11, NKIT12, NKIT17, NKIT19, NKIT20, NKIT25, NKIT31, NKIT34, and NKIT45), hypocotyls (NKIT1, NKIT2, NKIR6-9, NKIT14-16, NKIT18, NKIT21, NKIT26-29, NKIT35, NKIT37, NKIT41 and NKIT43), and cotyledon (NKIT5, NKIT22, NKIT23, and NKIT24), and other bacterial endophytic species were only exclusive to one or two tissues. However, three out of four species isolated from the V2 variety, namely, *B. safensis*, *B. australimaris*, and *Bacillus zhangzhouensis*, were found to inhabit all the three parts of tomato seedling such as root (NKIT46, NKIT51, NKIT56, NKIT64, NKIT67, NKIT68, and NKIT71), hypocotyls (NKIT49, NKIT50, NKIT55, NKIT59, NKIT60, NKIT62, NKIT63, NKIT66, and NKIT74), and cotyledon (NKIT47, NKIT48, NKIT52-54, NKIT57, NKIT58, NKIT70, NKIT72, NKIT73, and NKIT75), whereas, *Bacillus amyloliquefaciens* (strain NKIT61) was found only in the root region. According to BLAST analysis of sequenced 16S rRNA gene homology and phylogenetic analysis using the NJ approach with 100 bootstrap sampling, strain NKIT9 shared 99.16% identity with a variety of *B. siamensis* strains in the NCBI database. A phylogenetic tree based on 16S rRNA gene sequences was drawn, and it clearly showed strain NKIT9 as *B. siamensis* (Figure 2). Moreover, the endospore staining confirmed that it belongs to *Bacillus* genus (Supplementary Figure 1).

### Distribution, Diversity, and Richness of Bacterial Endophytes

**FIGURE 3:**
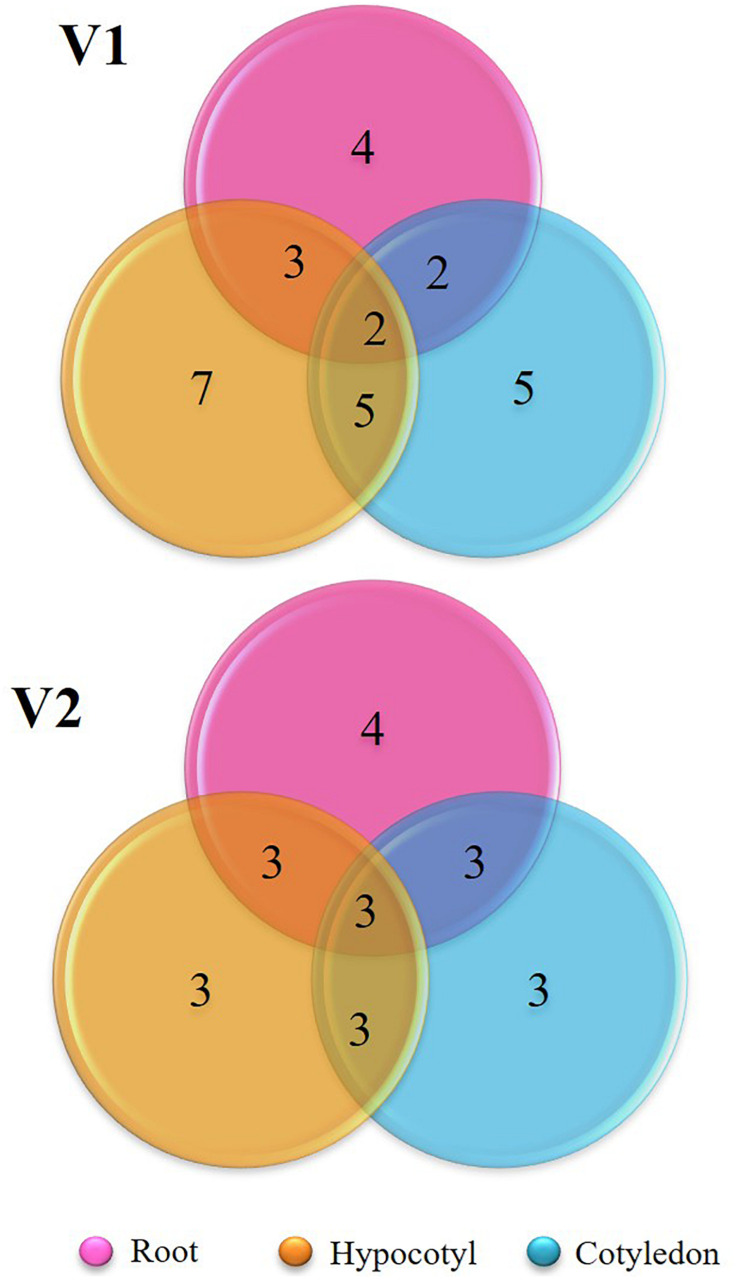
Venn diagram representing the shared species of isolated bacterial endophytes within the variety.

Diversity indices calculated between the bacterial endophytes isolated from each tissue of the two varieties of tomato plants used in the study are shown in Table 1. Shannon diversity (H’) was maximum in the cotyledon (1.56) and hypocotyls (1.34) of V1 variety. Least diversity was reported in the root region of V1 variety (0.83). Simpson’s index of diversity was maximum in the cotyledon (0.93) of V1, followed by root (0.77) of V2. Species richness was determined by counting the number of species in each group and was found at its maximum in hypocotyl (*n* = 7) of V1 variety followed by the cotyledon (*n* = 5) and root (*n* = 4) of V1. Magalef’ index, calculated to estimate the evenness between the species of both the types, was found to be highest in cotyledon (2.23) of V1 variety. Species shared between V1 and V2 were highest in hypocotyl, resulting in a high value of Sorenson’s similarity index (0.37) (Table 2). A Venn diagram illustrated the species’ number and the relationship between the isolated species within the same variety (Figure 3). Interestingly, the V1 variety of tomato (Pusa Ruby) contains a more diverse population of endophytic bacteria as compared to V2 (Table 1).

**TABLE 1:** Species diversity analysis of the bacterial isolated from V1 and V2 variety of tomato.

	V1			V2		
	Hypocotyl	Root	Cotyledon	Hypocotyl	Root	Cotyledon
Shannon diversity	1.34	0.83	1.56	0.93	1.28	0.91
Simpson’s index	0.34	0.54	0.06	0.36	0.22	0.38
Simpson’s index of diversity	0.65	0.45	0.93	0.63	0.77	0.62
Margalef’ index	1.82	1.20	2.23	0.91	1.30	0.83
Evenness	0.68	0.59	0.96	0.85	0.92	0.83
Species richness	7.00	4.00	5.00	3.00	4.00	3.00

**TABLE 2:** Comparison of different similarity indices among various regions of two organic varieties of tomato.

V1 vs. V2	Species shared	Jaccard’s SI	Sorensen’s SI
Root	1.00	0.10	0.20
Hypocotyl	3.00	0.23	0.37
Cotyledon	2.00	0.20	0.33

### Antifungal Activity of the Isolated Endophytic Bacteria

**FIGURE 4:**
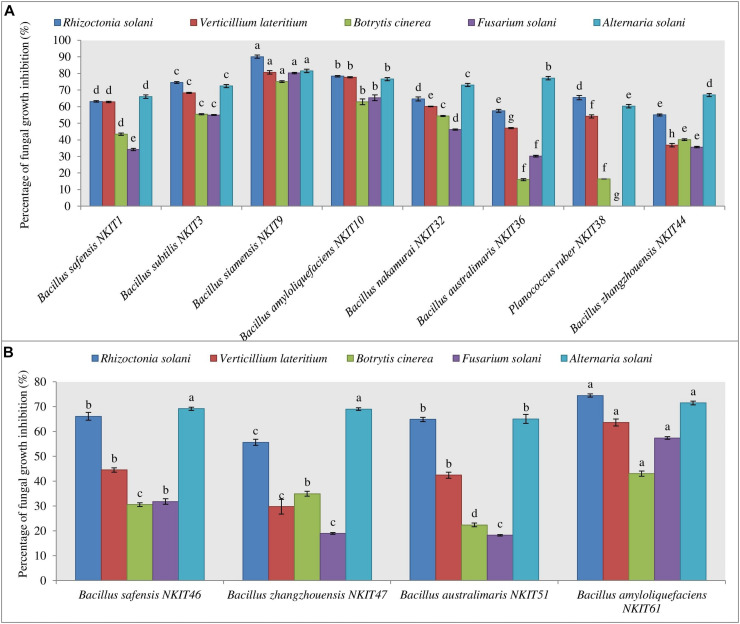
Antagonistic effect against five pathogenic test fungi. **(A)** Graph for the antifungal activity of bacterial endophytes isolated from V1 variety seeds. **(B)** Graph for the antifungal activity of bacterial endophytes isolated from V2 variety seeds. Three replicates were used for the assay. Bars labeled with the same letters are not significantly different according to Duncan Multiple Range Test at *p* = 0.05. Vertical lines represent the standard errors of the mean.

**FIGURE 5:**
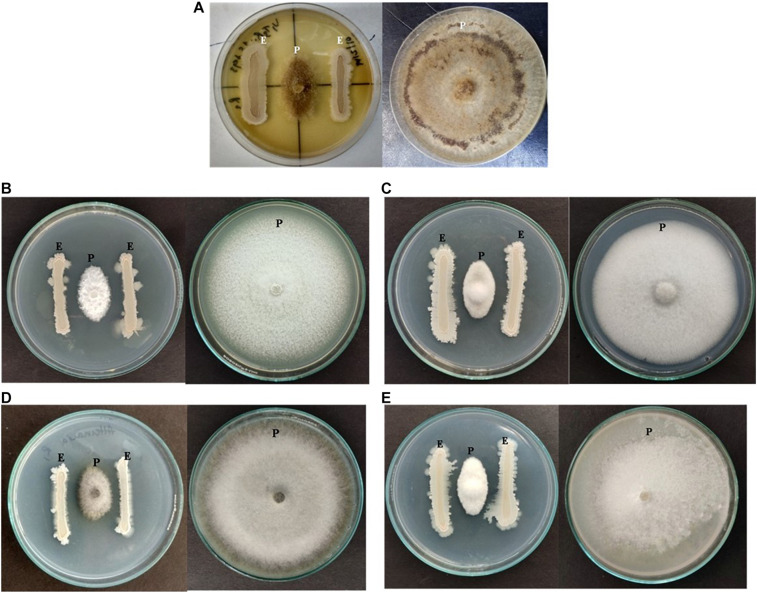
Antagonizing effect of strain NKIT9 against **(A)** *Rhizoctonia solani*, **(B)** *Verticillium lateritium* **(C)** *Botrytis cinerea*, **(D)** *Alternaria solani*, and **(E)** *Fusarium solani* after 6 days of inoculation (“E” represents endophytic bacterial strain whereas “P” represents Pathogenic fungi).

All the bacterial endophytes isolated from the two organic tomato varieties were screened for their antifungal activity against five economically important fungal pathogens of tomato crop viz. *R. solani, V. lateritium*, *B. cinerea, A. solani*, and *F. solani* through dual culture assay (Figures 4A,B and Supplementary Tables 2, 3). The dual culture bioassay’s key purpose was based on a bio-prospecting strategy to select potential endophytes with antifungal activity. Among all the isolates of V1, strain NKIT9 exhibited the highest antifungal activity with percentage growth inhibition values ranging from 75-90%, against all the five major pathogens of the tomato crop (Figure 5). The zone of inhibition persisted for up to 7 days of inoculation. To the best of our knowledge, this is the first report on the antifungal activity of endophytic *Bacillus siamensis* strain NKIT9 isolated from the organic varieties of tomato.

*B. amyloliquefaciens* strain NKIT10 was found to be the next best species. The activity pattern of *B. safensis* varied from strain to strain. The most active strain of *B. safensis viz. B. safensis* strain NKIT46 isolated from variety V2 recorded >70% growth inhibition activity against *R. solani* and *A. solani.* Simultaneously, strains of *B. australimaris*, *B. nakamurai*, and *B. zhangzhouensis* showed very low to nil activity against the selected pathogens. Heatmap dendrogram revealed that the antifungal activity of the tested strains against *R. solani* positively correlated with *A. solani* while activity against *B. cinerea* correlated with activity against *F. solani*. It shows the antagonism between the test fungi and the isolated bacterial strain of V1 and V2 (Supplementary Figure 2). The z-score clustering facilitates the bacterial species relationship between the isolates in relation to the antifungal activity.

The endophytic population from variety V1 has been observed to be more antagonistic against all the five pathogenic fungi than the V2. None of the endophytes were found active against all the five test pathogens. More than 68% of endophytic bacteria of V1 suppressed the growth of *R. solani* in the dual culture assay with antagonistic activity up to 90%. Meanwhile, 15% of its population showed the antagonistic effect against all the test pathogens with over 60 or more than 60% inhibition.

### Antifungal Activity of Extracted Lipopeptide

**FIGURE 6:**
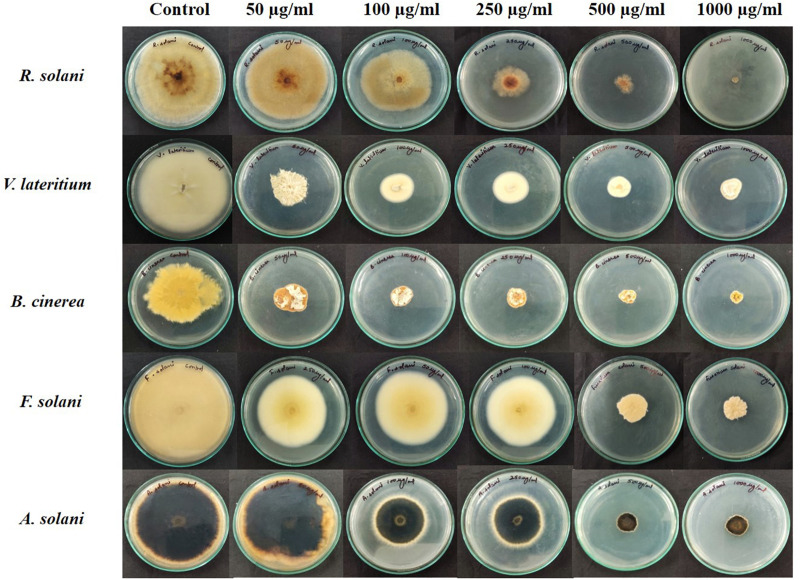
Antifungal bioassay of lipopeptide extracted from *Bacillus siamensis* strain NKIT9 at 5 different concentrations of 50, 100, 250, 500, and 1000 μg/ml against five test pathogenic fungi (*R. solani, F. solani, B. cinerea, A. solani*, and *V. lateritium).*

In this study, the results showed that the lipopeptide extract of the bacterial strain NKIT9 inhibited the growth of all test fungi with an IC_50_ value of 230, 276.2, 346.6, 470.5, and 329.9 μg/ml against *R. solani, V. lateritium, B. cinerea, F. solani*, and *A. solani*, respectively. A dose response with an *R*^2^ value of 0.867 and 100% growth inhibition of *R. solani* was observed at 1000 μg/ml extract of NKIT9 (Figure 6). The IC_50_ values were obtained using regression equation (Supplementary Figures 3A–E). The lipopeptide extract of strain NKIT9 was found to be most active against *R. solani* as compared to other test fungi with an IC_50_ value of 230 μg/ml. Based on the results of the dual culture assay and lipopeptide bioassay, *R. solani* was selected as the test fungi for *in vivo* tests for damping-off disease control and green house trial.

### Lipopeptide Profiling by UPLC-HDMS

**FIGURE 7:**
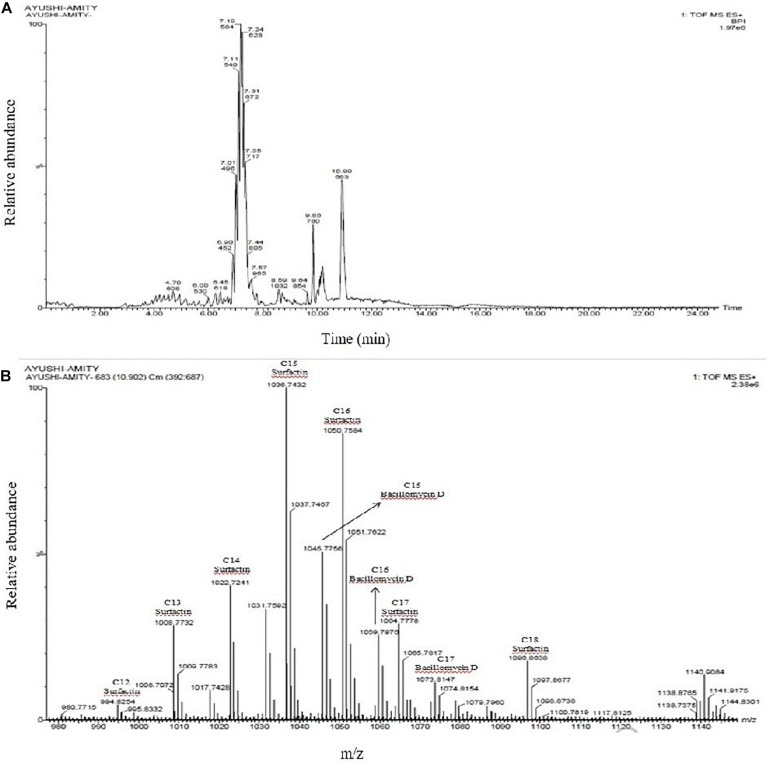
**(A)** UPLC chromatogram of lipopeptides extracted from *Bacillus siamensis* strain NKIT9; **(B)** HDMS accurate mass revealed the production of Surfactin and Bacillomycin D analogues.

**FIGURE 8:**
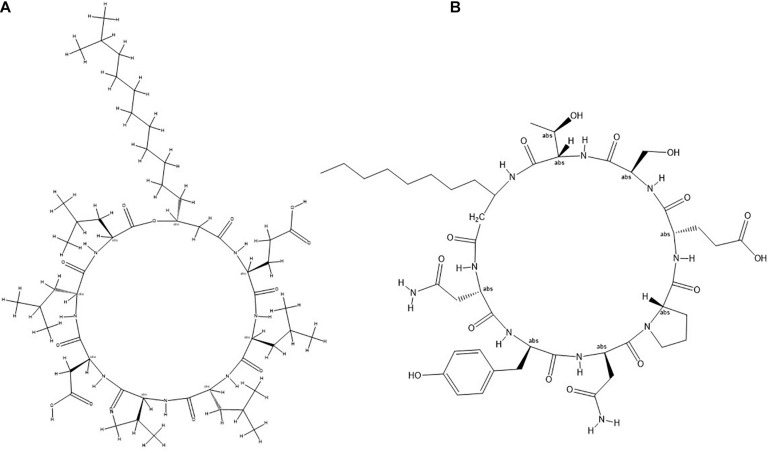
General molecular structure of lipopeptides **(A)** Surfactin and **(B)** Bacillomycin isolated from *B. siamensis* strain NKIT9.

To identify the lipopeptides present in the extract obtained from the bacterial strain NKIT9, Chromatographic separation and Mass Spectrometry of lipopeptide extract was performed on UPLC-H class with a Synapt G2-Si-High Definition Mass Spectrometry (HDMS) system. The UPLC total ion chromatogram of the bacterial extract is shown in Figure 7A. Figure 7B reveals the mass spectrum of the analyte showing the presence of the molecular peaks at m/z 994.8, 1008.77, 1022.72, 1036.74, 1050.75, 1064.77, 1096.86, 1045.77, 1059.79, and 1079.81. These masses were assigned to C12 Surfactin [M + H+]+, C13 Surfactin [M + H+]+, C14 Surfactin [M + H+]+, C15 Surfactin [M + H+]+, C16, Surfactin [M + H+]+, C17 Surfactin [M + H+]+, linear C18 Surfactin and, C15 Bacillomycin D [M + H+]+ respectively (Supplementary Table 4). The general molecular structures of the isolated antifungal lipopeptides are presented in Figure 8.

### Damping-Off Assay

**FIGURE 9:**
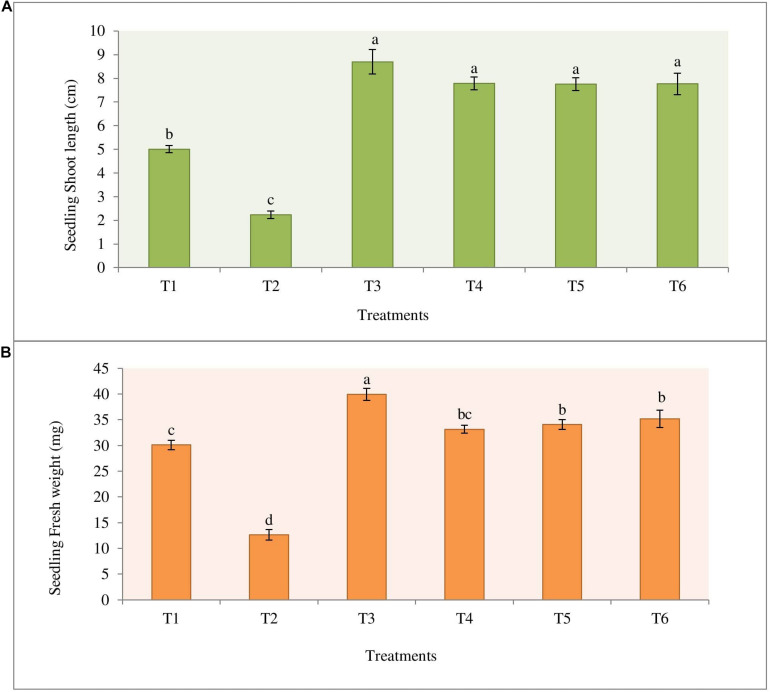
Effect of priming of tomato seeds with strain NKIT9 on the **(A)** shoot length of the seedling (*F* = 54.109; df = 53) and **(B)** seedling fresh weight (*F* = 70.928; df = 53). Evaluation was made after 15 days of planting of tomato seeds. Nine replicates were used for the assay with 10 seedlings in each replicate. Bars labeled with the same letters are not significantly different according to Duncan Multiple Range Test at *p* = 0.05. Vertical lines represent the standard errors of the mean.

The highest percentage of germination (96.7%) was observed in the treatment T3 (Bio-primed seeds) with no diseased plant. While the highest percentage of the diseased (72.2%) seedlings was observed in the treatment T2 (Surface sterilized seeds treated with fungal pathogen). Desiccation of the roots was observed in germinated seeds and young seedlings due to damping-off symptoms (Supplementary Figure 4). Treatments 4 (Bio-primed seeds + fungal pathogen) and T5 (Bio-primed seeds + fungal pathogen + soil treatment of the bacterial strain NKIT9) observed the second best treatment after T3 with only 28.9% and 27.8% diseased plants, respectively (Table 3). Chemical treatment was found to be less effective than the introduced bio-control agent in the control of damping-off disease of tomato seedlings. Compared with the positive control, the seed treatment with strain NKIT9 enhanced the seedling shoot length from 2.23 cm to 7.79 cm (Figure 9A) and seedling fresh weight from 12.6 mg to 33.2 mg and (Figure 9B). These findings suggest that strain NKIT9 can help in protecting tomato seedlings from damping-off and promoting the plant growth.

**TABLE 3:** Effects of strain NKIT9 on seedling germination, the suppression of damping-off of tomato plants caused by *R. solani* 15 days after planting*.

Treatments	Seedling germination (%)	Diseased plants %
T1: Surface- sterilized seeds only (control)	74.44 ± 0.17c	0 ± 0.00d
T2: Surface-sterilized seeds + fungal pathogen	48.89 ± 0.26d	72.22 ± 0.40a
T3: Bio–primed seeds only (for growth promoting activity)	96.67 ± 0.16a	0 ± 0.00d
T4: Bio- primed seeds + fungal pathogen	81.11 ± 0.20b,c	28.89 ± 0.42c
T5: Bio primed seeds + fungal pathogen + soil treatment of the bacterial strain NKIT9	82.22 ± 0.27b	27.78 ± 0.46c
T6: Chemical treated seeds (Bavistin-Carbendazim) + fungal pathogen (chemical control)	87.78 ± 0.30b	42.22 ± 0.40b

*For each treatment, each datum is an average of results of nine replicates with ± SE. Means in any column with different letters are significantly different (*P* = 0.05) according to Duncan Multiple Range Test with *F*-value of 42.387 and 61.938 for seedling germination and disease percentage. Whereas, “a” signifies highest value followed by “b” > “c” > “d.”

### Pot Experiment

**FIGURE 10:**
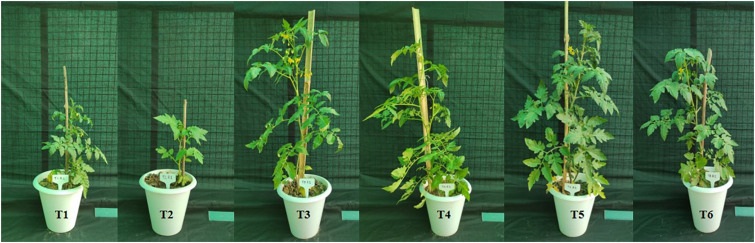
Growth of tomato plants (V1) obtained after 45 days of sowing of tomato seeds of all the treatments: T1 (Surface-sterilized seeds only); T2 (Surface-sterilized seeds + fungal pathogen); T3 (Bio-primed seeds only); T4 (Bio-primed seeds + fungal pathogen); T5 (Bio-primed seeds + fungal pathogen + soil treatment of the bacterial strain NKIT9); T6 (Chemical treated seeds (Bavistin-Carbendazim) + fungal pathogen.

Under greenhouse conditions, the strain NKIT9 showed strong control of *R. solani.* According to the results of plant growth promotion treatment, *B. siamensis* strain NKIT9 had a noticeable effect on tomato plant growth in a greenhouse. Interestingly bio-primed seeds treated with fungal pathogen (T4) recorded highest yield, plant height, buds, flowers, and fruits followed by T3 and T5 (Table 4). Fungicide treated seeds recorded yield comparable to T2. The plant height increased by 58.9% and the number of flowers increased by 62.2% as compared to T2 (Figure 10). T5 treatment plants showed statistically similar growth patterns compared to the chemical control (T6). These findings suggested that the strain NKIT9 could successfully control the *R. solani in vivo* and could be used as a bio-control agent.

**TABLE 4:** Morphological characters and fruit yield of tomato crops observed with different treatments.

Treatments	Measure of physical parameters per plant				
	Plant height (cm)	No. of buds	No. of flowers	No. of fruits (70 DAP*)	Fruit yield (g)
T1: Surface- sterilized seeds only (control)	20.09 ± 2.74d	4.63 ± 1.22a–c	11.35 ± 1.57b,c	5.56 ± 3.43b,c	500.62 ± 71.25b,c
T2: Surface-sterilized seeds + fungal pathogen	20.93 ± 3.43d	1.06 ± 0.34d	8.19 ± 1.65c	4.78 ± 3.43c	430.31 ± 90.55c
T3: Bio–primed seeds only (for growth promoting activity)	46.94 ± 1.98a,b	3.38 ± 0.49b–d	15.81 ± 2.25b	7.90 ± 3.43b	711.56 ± 101.39b
T4: Bio- primed seeds + fungal pathogen	51.00 ± 1.90a	7.06 ± 0.98a	23.44 ± 2.73a	11.72 ± 3.43a	1054.69 ± 123.02a
T5: Bio primed seeds + fungal pathogen + soil treatment of the bacterial strain NKIT9	42.28 ± 1.54b	4.63 ± 1.02a–c	16.93 ± 1.86b	8.46 ± 3.43b	762.19 ± 83.73b
T6: Chemical treated seeds (Bavistin-Carbendazim) + fungal pathogen (chemical control)	23.75 ± 3.41c	5.50 ± 1.06a,b	9.06 ± 1.45d	4.53 ± 3.43c	407.81 ± 65.40c
*F*-value	*18.99*	*4.26*	*6.87*	*7.42*	*7.42*

*Values are mean of 16 replications ± SE. Means followed by a common letter are not significantly different at 5% level by DMRT. Whereas, “a” signifies highest value followed by “b” > “c” > “d.” DAP, Days after planting.*

## Discussion

The present research explores two organic tomato varieties for diversity of endophytic bacteria and their antifungal ability against selected fungal pathogens. This is the first report on the diversity study of endophytic bacteria from organic tomato plants. The seedlings of the V1 tomato plant variety were found to have high species abundances and the diversity of bacterial endophytes. The plausible reason for the disparity in endophyte diversity between the two tomato plant varieties could be the variations in the rhizospheric microbiome that probably contribute to differential bacterial colonization in the plant endosphere (Compant et al., 2010; Liu et al., 2017). Species richness was found at its maximum in the hypocotyl of the seedling (*n* = 7) of V1. Evenness was found at its maximum in V1 cotyledon (0.96). A value for evenness approaching zero reflects large differences in the abundance of species, whereas, an evenness of one means all species are equally abundant. These findings indicate that endophytic bacteria can exhibit a tissue-specific distribution, which has also been reported from other systems (Reinhold-Hurek and Hurek, 2011; Thomas and Reddy, 2013; Xia et al., 2015). Previous studies have shown the species specificity of endophytes. The difference in endophytic assemblies in different tissue types can be due to the difference in their potential to use the substrate (Huang et al., 2008; Chowdhary and Kaushik, 2015). Yang et al. (2011) reported 72 bacterial endophytes, including 45 from the stem and 27 from the healthy tomato plant leaves, and found *Brevibacillus brevis* W4, an endophyte antagonistic to *B. cinerea*. We believe that different agro-climatic locations (V1 from Maharashtra and V2 from Andhra Pradesh) resulted in endophytic population variations in the current study. The bacterial strains isolated from the tomato seedlings must contain the same phylum of the bacterial population as those present in seeds (López et al., 2018). These findings indicate that tomato seeds can comprise a fundamental group of bacteria that are likely to reach seeds during their reproductive development, and play specific roles in seed or seedling growth (López et al., 2018). Firmicutes, the phylum that mostly colonizes seeds, grew faster in seedlings, implying that seed germination offers a nutritional benefit that boosts this group’s development. Some of the bacteria found in seeds, such *as Bacillus*, are known to produce endospores, which may explain their high occurrence in seeds (López et al., 2018). The host genotype is reported to play an essential role in managing the associated plant microorganisms, particularly the endophytes (Lundberg et al., 2012; Podolich et al., 2015; Upreti and Thomas, 2015). Also, there are indications of endophytic bacterial transmission via seeds, which might clarify their possible integral interaction with a specific host varietal (Truyens et al., 2014). Seeds or associated fruits are ecologically important as units of dispersal, succession, invasion, and survival in the face of adversity. Germination causes fierce competition among seedlings, which shapes wild communities and necessitates management in crop situations (Newcombe et al., 2018).

Despite being identical in the presence of species, our findings show that under regulated conditions, not all bacteria inhibit mycelial growth; however, they vary in their ability to synthesize other inhibitory molecules. In comparison to the endophytes in variety V2, the V1 endophytic population is increasingly antagonistic to all five test fungi. The most potent antagonistic endophyte was identified through 16S rRNA gene sequencing as *B. siamensis* NKIT9. There was no physical contact between the isolates and the pathogen in the inhibition zone, indicating that the isolated active *Bacillus* species may generate definite antifungal substances that impede the mycelial growth (Lee et al., 2008). *B. siamensis* strain NKIT9 exhibits more antagonistic activity than other species against all the selected fungal pathogens. The *z*-score clustering facilitates the bacterial species relationship between the isolates in relation to the antifungal activity. A higher *z*-value suggests that genotypes will be better clustered by function, suggesting a clustering result, which is more biologically important (Bhattacharya et al., 2012). In variety V1, *B. cinerea and F. solani* are less susceptible to antifungal behavior of some endophytic species or have similar responses to most of the bacterial species. Likewise, *R. solani* and *A. solani* linked similar responses with those of *V*. *lateritium.* This clustering is not by chance but because of the similarity in antifungal activity. However, in variety V2, *B. amyloliquefaciensis* was notable for maximum antifungal activity against all pathogenic fungi.

Frequent and excessive use of fungicides has led to the development of the pathogen’s resistant races (Brent and Hollomon, 2007), such as 2-amino-pyrimidineresistant *Blumeria graminis* causing powdery mildew (Brent, 1982), phenylamides resistant *Phytophthora infestans* to (Staub, 1994), Melanin Biosynthesis Inhibitors (Dehydratase) MBI-D resistant rice blast causing *Magnoprthe grisea* (Kaku et al., 2003). Due to the appearance of these resistant strains and rising environmental contamination, seed bio-priming with endophytes is being looked upon as an environmentally friendly option. It has been used as an alternative method for controlling a variety of seed and soil-borne pathogens in recent years. It is an ecological strategy that employs selected fungal antagonists to combat pathogens found in soil and seeds (Reddy, 2012). Seed bio-priming improved the effectiveness of *B. subtilis*, in the control of root rot pathogens (*F. solani* and *R. solani*) in greenhouse trials, with bio-priming seed treatments showing the highest percentages of disease reduction (Reddy, 2012). Many horticultural crops used seed priming as a tool to improve germination speed and uniformity, as well as final stand (Bisen et al., 2015). However, if seeds are infected or damaged with infectious agents, fungal growth can be accelerated during priming, causing negative plant effects (Reddy, 2012). In the study conducted by El-Mohamedy and Abd El-Baky (2008), after 15, 45, and 60 days of sowing of bio-primed pea seeds, the disease incidence reduced by 72.7–84.5, 72.2–82.9, and 67.6–80.0%, respectively, at the pre- and post-emergence stages. Seed priming, either alone or in combination with low-dose fungicides and/or bio-control agents, is being used to improve seed emergence rate and uniformity while reducing damping-off disease (Reddy, 2012). Therefore, formulation or suspension of biological agent can also be used as a booster dose in plant protection. Many endophytic strain treatments of tomato seeds, namely *Rhizobium taibaishanense* (RBEB2), *Pseudomonas psychrotolerance* (REB4), *Microbacterium testaceum* (RBEB1), and *Bacillus subtilis* (RBEB6), have previously shown significantly improved seed germination, seedling growth, vigor index, and biomass production (Karthik et al., 2017). In the present study, the bio-primed tomato seeds showed reduced percentage of damping-off diseased plants when challenged with *R. solani* as compared to the control indicating *B. siamensis* strain NKIT9 treatment exhibited a significant disease resistance (*p* < 0.05). Under greenhouse conditions also, the strain NKIT9 suppressed *R. solani* infestation and showed increase in fruit yield by 59.2% compared with the untreated *R. solani* infested control. Increased fruit yield concomitantly led to low disease incidence of *R. solani* in tomato plants. This plant growth promoting properties of endophytic strains makes them a more suitable alternative for chemical agents.

Studies have reported the production of wide variety of structurally different antagonistic secondary metabolites from many endophytic and non-endophytic *Bacillus* spp. including *B. siamensis* (Fira et al., 2018). Interestingly, the strains producing non-ribosomally synthesized lipopeptides and peptides have shown enhanced fungicidal activities (Etchegaray et al., 2008; Dimkiæ et al., 2013). The LC-MS/MS-based analysis of the strain NKIT9 extract further confirmed the product of surfactin derivatives, iturin, and fengycin by *Bacillus* sp. (Jasim et al., 2016). The current study shows the first experimental evidence of the presence of these antifungal lipopeptides in *B. siamensis* isolated from a tomato variety. The IC_50_ value of 230 μg/ml showed the high potency of the crude lipopeptide extract obtained from the pure culture of *B. siamensis* strain NKIT9 to inhibit the growth of *R. solani*, thus, further confirming that the antifungal activity of the *B. siamensis* is due to lipopeptides. Earlier, it was predicted through genome sequencing that *B. siamensis* contains Sufactin and Bacillomycin D genes (Pan et al., 2019). However, in the current study, this is the first time that it has been extracted and confirmed in the culture broth. It is believed that bioactive compounds producing bacterial endophytes can be an effective biological agent and a powerful tool for the development of a formulation against fungal pathogens in crop protection and for promoting plant growth. The mechanism of action of lipopeptides might depend on the structural and functional properties of lipopeptides (Zhang et al., 2013). Bacillomycin L antifungal activity against *R. solani Kühn*, which includes a specific association with intact fungal hyphae, has been extensively investigated using different fluorescent methods, gel retardation experiments, and electron microscopy (Zhang et al., 2013).

Previously, *B. siamensis* strain isolation has been reported from rhizosphere or other sources other than endophytic (Hussain and Khan, 2020; Yoo et al., 2020; Pastor-Bueis et al., 2017; Islam et al., 2019). Recently, Putri et al., 2020, showed antifungal activity of ethyl acetate extract obtained from fermentation filtrate of *B. siamensis* against *Aspergillus niger.* Various *Bacillus* strains, including *B. siamensis*, were previously identified with surfactin gene and produce surfactin like biosurfactants (Mehetre et al., 2019). A similar study by Pan et al. (2019) reported that *B. siamensis* produced sets of bacillibactins, fengycins, bacillomycins, and surfactins through the mining of genome and metabolic profiling. Another PCR based study identified the genes, namely surfactin synthetase D, and bacillomycin synthetase D, involved in cyclic lipopeptide biosynthesis against multidrug-resistant aquatic bacterial pathogens (Xu et al., 2018). Interestingly, no endophytic strain of *B. siamensis* isolated from a tomato variety with antifungal potential has been reported to produce surfactin and Bacillomycin D. Complete isolation and identification of these lipopeptides from *B. siamensis* strain NKIT9 isolated from the varieties of tomato plants. Overall, this indicates that the use of bacteria native to host plants may increase the success rate in screening bio-control agents because these isolates are likely to be better adapted to the host and its associated environmental conditions than other strains selected from culture collections (Köberl et al., 2013; Karimi et al., 2016).

## Conclusion

The data presented here collectively support the notion that soil properties and rhizospheric microflora can affect the endophytic microflora. To the best of our knowledge, this is the first report of the isolation and diversification of bacterial endophytes from organic tomato seeds; however, we only found the presence of *Bacillus* species. Comparatively, Pusa Ruby has a more diverse and biologically active endophytic population of bacteria. Results demonstrated that *Bacillus* sp. strain NKIT9 showed the highest *in-vitro* antifungal activity against *R. solani*, which produces bioactive lipopeptide compound as identified through Ultra Performance Liquid-Chromatography High Definition Mass Spectrometry (UPLC-HDMS). In addition, in vitro findings revealed that the strain NKIT9 significantly reduced disease incidence which makes this strain a promising antifungal bio-control agent.

## Data Availability Statement

The datasets presented in this study can be found in online repositories. The names of the repository/repositories and accession number(s) can be found in the article/Supplementary Material.

## Author Contributions

NK and ND conceived the idea, secured funding, planned the work, and guided the AyS. AyS performed the experimental work and wrote the manuscript. AbS helped in data analysis. NK, AbS, and ND read and reviewed the manuscript. AB, MR, and YS conducted the molecular identification of all the bacterial isolates. TM supported in lipopeptide extraction process. All authors contributed to the article and approved the submitted version.

## Conflict of Interest

The authors declare that the research was conducted in the absence of any commercial or financial relationships that could be construed as a potential conflict of interest.

## Footnotes

1 www.heatmapper.ca
