# Supplementary material for: Screening of Tomato Seed Bacterial Endophytes for Antifungal Activity Reveals Lipopeptide Producing Bacillus siamensis Strain NKIT9 as a Potential Bio-Control Agent
Source: Front Microbiol. 2021 Jun 10;12:609482. doi: 10.3389/fmicb.2021.609482 (PMC8222588; doi:10.3389/fmicb.2021.609482)
Supplement: Supplementary file 1 [file Data_Sheet_1.docx]

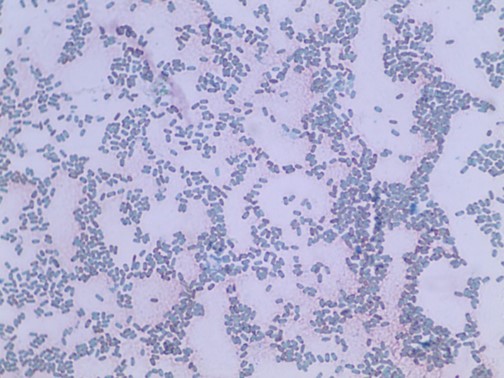


**FIGURE S1.** Endospore staining, using malachite green as a primary stain, confirmed the production of endospores by *B. siamensis* strain NKIT9


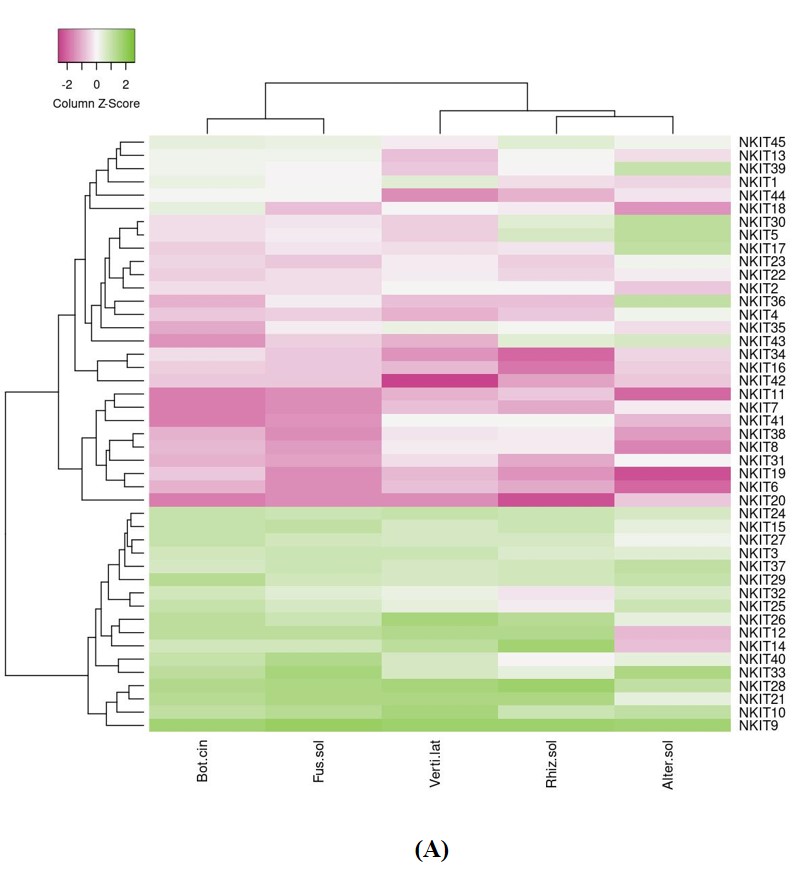


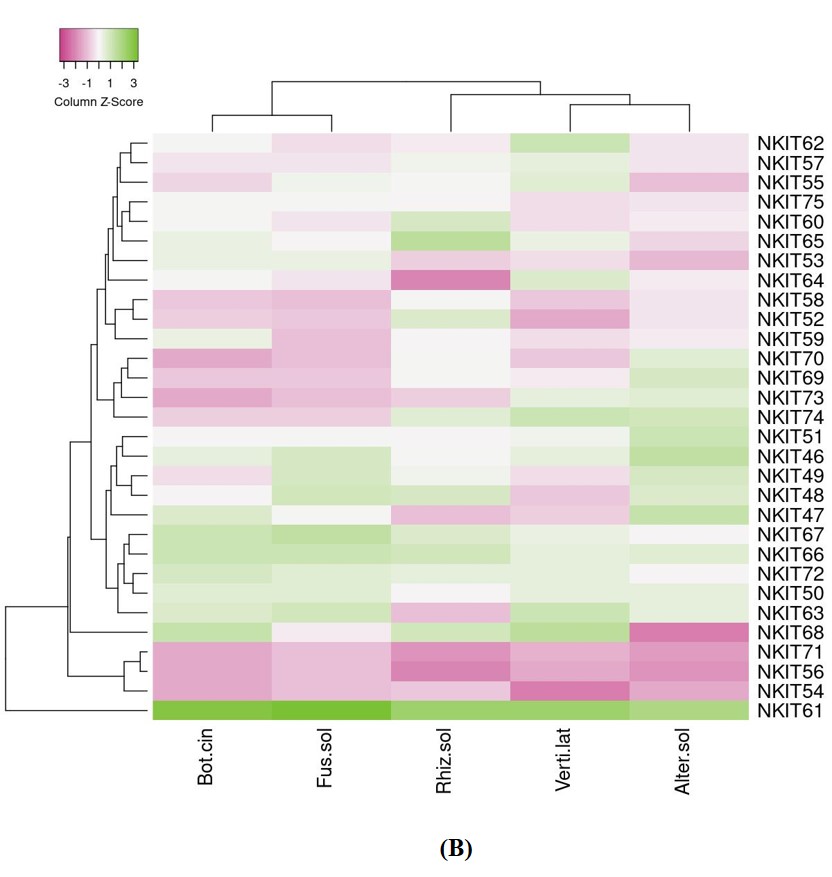


**FIGURE S2.** Heatmap illustrating the strength of antifungal activity of different species of bacterial strains of (A) V1 and (B) V2 against all five pathogenic fungi with respect to each other (Fungal pathogens mentioned in sequence: *Botrytis cinerea*; *Fusarium solani; Verticillium lateritium; Rhizoctonia solani*; *Alternaria solani*)

**(A)**

**(B)**

**(C)**

**(D)**

**(E)**

**FIGURE S3.** Inhibition percentage of lipopeptide extract against (A) *R. solani;* (B) *V. lateritium;* (C) *B. cinerea* ; (D) *F. solani* and (E) *A. solani*. IC_50_ value was calculated using regression equation


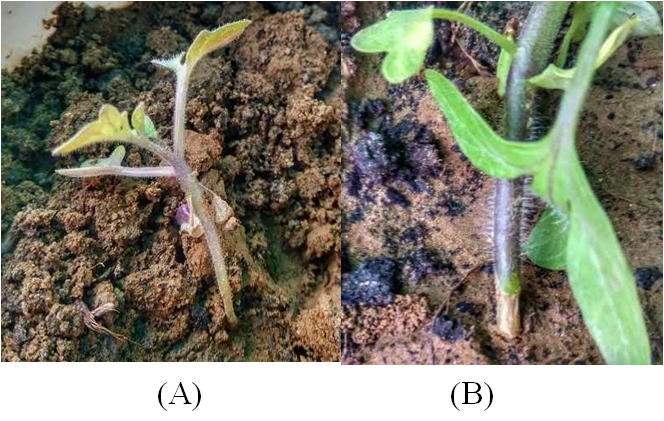


**FIGURE S4.** Diseased roots of tomato seedlings in positive control causing damping-off due to *R. solani*
