# Supplementary material for: Screening of Tomato Seed Bacterial Endophytes for Antifungal Activity Reveals Lipopeptide Producing Bacillus siamensis Strain NKIT9 as a Potential Bio-Control Agent
Source: Front Microbiol. 2021 Jun 10;12:609482. doi: 10.3389/fmicb.2021.609482 (PMC8222588; doi:10.3389/fmicb.2021.609482)
Supplement: Supplementary file 2 [file Data_Sheet_2.docx]

**TABLE S1.** Bacterial isolates investigated in this study were identified based on 16S rRNA gene sequence similarity comparison using *blastn* and *nr/nt* database. Details of seventy-five strains belonging to 8 representative bacterial species^1^ including source of isolation, percent similarity, and NCBI nucleotide submission accession are mentioned

| No. of OTUs | Strains | Source | Accession number | % Identity to NCBI closet match^2^ |
| --- | --- | --- | --- | --- |
| 07 | *Bacillus safensis* FO-36b (T) (MK424279.1) | | | |
|  | NKIT1 | V1 | MW586044 | 99.17 |
|  | NKIT4 |  | MW586047 | 99.24 |
|  | NKIT7 |  | MW586050 | 99.17 |
|  | NKIT8 |  | MW586051 | 99.19 |
|  | NKIT15 |  | MW586058 | 99.15 |
|  | NKIT16 |  | MW586059 | 99.19 |
|  | NKIT22 |  | MW586065 | 99.12 |
| 18 | *Bacillus safensis* FO-36b (CP010405.1) | | | |
|  | NKIT2 | V1 | MW586045 | 99.16 |
|  | NKIT5 |  | MW586048 | 99.14 |
|  | NKIT6 |  | MW586049 | 99.08 |
|  | NKIT11 |  | MW586054 | 99.14 |
|  | NKIT17 |  | MW586060 | 99.13 |
|  | NKIT18 |  | MW586061 | 99.26 |
|  | NKIT19 |  | MW586062 | 99.14 |
|  | NKIT20 |  | MW586063 | 99.21 |
|  | NKIT23 |  | MW586066 | 99.14 |
|  | NKIT25 |  | MW586068 | 99.25 |
|  | NKIT27 |  | MW586070 | 99.21 |
|  | NKIT30 |  | MW586073 | 99.14 |
|  | NKIT31 |  | MW586074 | 99.19 |
|  | NKIT35 |  | MW586078 | 98.95 |
|  | NKIT37 |  | MW586080 | 99.18 |
|  | NKIT41 |  | MW586084 | 99.10 |
|  | NKIT43 |  | MW586086 | 98.94 |
|  | NKIT45 |  | MW586088 | 99.03 |
| 12 | *Bacillus safensis* strain NBRC 100820 (NR_113945.1) | | | |
|  | NKIT34 | V1,V2 | MW586077 | 98.85 |
|  | NKIT46 |  | MW586089 | 98.93 |
|  | NKIT48 |  | MW586091 | *98.34* |
|  | NKIT49 |  | MW586092 | 99.14 |
|  | NKIT50 |  | MW586093 | 99.16 |
|  | NKIT53 |  | MW586096 | 98.81 |
|  | NKIT54 |  | MW586097 | 99.26 |
|  | NKIT57 |  | MW586100 | *98.36* |
|  | NKIT60 |  | MW586103 | *98.51* |
|  | NKIT65 |  | MW586108 | 99.31 |
|  | NKIT66 |  | MW586109 | 99.28 |
|  | NKIT74 |  | MW586117 | 99.14 |
| 15 | *Bacillus australimaris* strain MCCC 1A05787 (NR_148787.1) | | | |
|  | NKIT36 | V1,V2 | MW586079 | *98.33* |
|  | NKIT39 |  | MW586082 | 98.91 |
|  | NKIT42 |  | MW586085 | *98.49* |
|  | NKIT51 |  | MW586094 | 98.73 |
|  | NKIT52 |  | MW586095 | 98.91 |
|  | NKIT55 |  | MW586098 | 98.76 |
|  | NKIT58 |  | MW586101 | 99.08 |
|  | NKIT59 |  | MW586102 | 99.15 |
|  | NKIT62 |  | MW586105 | 98.71 |
|  | NKIT68 |  | MW586111 | 98.93 |
|  | NKIT69 |  | MW586112 | 98.98 |
|  | NKIT70 |  | MW586113 | 98.87 |
|  | NKIT72 |  | MW586115 | 99.12 |
|  | NKIT73 |  | MW586116 | 98.93 |
|  | NKIT75 |  | MW586118 | 98.97 |
| 01 | *Bacillus amyloliquefaciens* DSM7 (FN597644.1) | | | |
|  | NKIT10 | V1 | MW586053 | 98.78 |
| 02 | *Bacillus amyloliquefaciens* strain MPA 1034 (NR_117946.1) | | | |
|  | NKIT33 | V1,V2 | MW586076 | 98.91 |
|  | NKIT61 |  | MW586104 | 98.87 |
| 01 | *Bacillus nakamurai* strain NRRL B-41091 (NR_151897.1) | | | |
|  | NKIT32 | V1 | MW586075 | *97.98* |
| 08 | *Bacillus siamensis* KCTC 13613(T) (KY643639.1) | | | |
|  | NKIT9 | V1 | MW586052 | 99.16 |
|  | NKIT12 |  | MW586055 | 99.16 |
|  | NKIT14 |  | MW586057 | 99.03 |
|  | NKIT21 |  | MW586064 | 99.16 |
|  | NKIT24 |  | MW586067 | 99.21 |
|  | NKIT26 |  | MW586069 | 99.20 |
|  | NKIT28 |  | MW586071 | 98.92 |
|  | NKIT29 |  | MW586072 | 99.14 |
| 07 | *Bacillus zhangzhouensis* strain MCCC 1A08372 (NR_148786.1) | | | |
|  | NKIT44 | V1,V2 | MW586087 | 99.10 |
|  | NKIT47 |  | MW586090 | 99.14 |
|  | NKIT56 |  | MW586099 | 99.15 |
|  | NKIT63 |  | MW586106 | 99.02 |
|  | NKIT64 |  | MW586107 | 99.18 |
|  | NKIT67 |  | MW586110 | 99.22 |
|  | NKIT71 |  | MW586114 | 99.11 |
| 01 | *Bacillus zhangzhouensis* DW5-4(T) (JOTP01000061) | | | |
|  | NKIT13 | V1 | MW586056 | 98.99 |
| 01 | *Bacillus subtilis* strain KCTC 13429 (CP029465.1) | | | |
|  | NKIT3 | V1 | MW586046 | *95.39* |
| 01 | *Bacillus subtilis subsp. subtilis* strain 168 (NR_102783.2) | | | |
|  | NKIT40 | V1 | MW586083 | 98.94 |
| 01 | *Planococcus ruber* strain CW1 (NR_157741.1) | | | |
|  | NKIT38 | V1 | MW586081 | *97.95* |

*^1^For queries which showed similarity with genomes of representative species mentioned in the table, the identity is confirmed by analyzing the alignment of query strain sequence with identified regions of the genomes and related Genbank file.*

*^2^For percent identity 95< and <98.6 to NCBI closet match, genus level identification is considered. (Kim et al 2014).*

Kim, M., Oh, H. S., Park, S. C., and Chun, J. (2014). Towards a taxonomic coherence between average nucleotide identity and 16S rRNA gene sequence similarity for species demarcation of prokaryotes. *Int. J. Syst. Evol. Microbiol.* 64, 346–351. doi: 10.1099/ijs.0.059774-0

**TABLE S2:** Inhibition percentage of isolated bacterial endophytes from V1 variety against plant pathogenic fungi

| S.No. | Bacterial endophytic strains (V1) | Percentage inhibition of fungal pathogens (%) | | | | |
| --- | --- | --- | --- | --- | --- | --- |
|  |  | ***Rhizoctonia Solani*** | ***Verticillium lateritium*** | ***Botrytis cinerea*** | ***Fusarium solani*** | ***Alternaria solani*** |
| 1. | *Bacillus safensis* NKIT1 | 63.1^m, n^ | 62.9^h^ | 43.4^j^ | 34.1^l, m^ | 66^n ,o, p^ |
| 2. | *Bacillus safensis* NKIT2 | 66.6 ^j ,k, l^ | 58.0^j, k^ | 29.9^m^ | 25.7^q, r^ | 64.2^q, r^ |
| 3. | *Bacillus subtilis* NKIT3 | 74.6^f, g, h^ | 68.3^e, f^ | 55.5^g^ | 55.0^f, g^ | 72.4^f, g, h^ |
| 4. | *Bacillus safensis* NKIT4 | 58.5^p, q^ | 44.7^q, r^ | 21.8^o^ | 20.2^s, t^ | 70.1^i, j^ |
| 5. | *Bacillus safensis* NKIT5 | 75.3^e, f^ | 49.4^o^ | 29.9^m^ | 31.2^n, o^ | 77.5^c, d, e^ |
| 6. | *Bacillus safensis* NKIT6 | 54.3^r^ | 46.2^p, q, r^ | 15.2^p, q^ | 0.0^w^ | 54.7^u^ |
| 7. | *Bacillus safensis* NKIT7 | 54.2^r^ | 46.7^p, q, r^ | 0.0^s^ | 0.0^w^ | 67.8^k, l, m^ |
| 8. | *Bacillus safensis* NKIT8 | 65.6^k, l^ | 55.2^l^ | 17.1^p^ | 4.1^v^ | 57.6^t^ |
| 9. | *Bacillus siamensis* NKIT9 | 90.1^a^ | 80.7^a^ | 75.1^a^ | 80.3^a^ | 81.5^a^ |
| 10. | *Bacillus amyloliquefaciens* NKIT10 | 78.3^d^ | 77.6^b, c^ | 62.8^e^ | 65.4^d^ | 76.6^c, d, e^ |
| 11. | *Bacillus safensis* NKIT11 | 58.6^p, q^ | 44.3^r^ | 0.0^s^ | 0.0^w^ | 54.8^u^ |
| 12. | *Bacillus siamensis* NKIT12 | 84.1^c^ | 75.7^c^ | 64.3^d, e^ | 61.6^e^ | 62.9^r^ |
| 13. | *Bacillus zhangzhouensis* NKIT13 | 68.4^j^ | 46.3^p, q, r^ | 42.4^j, k^ | 38.3^k^ | 66.3^m, n, o^ |
| 14. | *Bacillus siamensis* NKIT14 | 89.1^a^ | 72.7^d^ | 55.1^g^ | 51.6^h, i^ | 63.4^q, r^ |
| 15. | *Bacillus safensis* NKIT15 | 78.9^d^ | 66.1^f, g^ | 59.9^f^ | 59.8^e^ | 71.4^h, i^ |
| 16. | *Bacillus safensis* NKIT16 | 45.5^t^ | 45.3^q, r^ | 26.0^n^ | 19.6^s, t^ | 64.9^o, p, q^ |
| 17. | *Bacillus safensis* NKIT17 | 64.3^l, m^ | 52.8^m, n^ | 25.9^n^ | 27.9^p, q^ | 77.0^c, d, e^ |
| 18. | *Bacillus safensis* NKIT18 | 65.9^k, l^ | 56.6^k, l^ | 45.6i | 16.3^u^ | 59.5^s^ |
| 19. | *Bacillus safensis* NKIT19 | 50.4^s^ | 45.8^q, r^ | 23.1^o^ | 0.0^w^ | 52.5^v^ |
| 20. | *Bacillus safensis* NKIT20 | 38.8^v^ | 36.8^s^ | 0.0^s^ | 0.0^w^ | 64.7^p, q^ |
| 21. | *Bacillus siamensis* NKIT21 | 86.3^b^ | 77.1^b, c^ | 67.6^c^ | 69.7^c^ | 71.4^h, i^ |
| 22. | *Bacillus safensis* NKIT22 | 61.9^n, o^ | 54.9^l^ | 26.0^n^ | 25.0^r^ | 68.4^k, l^ |
| 23. | *Bacillus safensis* NKIT23 | 60.0^o, p^ | 54.6^l, m, n^ | 28.2^m^ | 19.6^s, t^ | 70.2^i, j^ |
| 24. | *Bacillus siamensis* NKIT24 | 78.9^d^ | 70.0^e^ | 60.0^f^ | 55.7^f^ | 73.6^f^ |
| 25. | *Bacillus safensis* NKIT25 | 65.2^k, l ,m^ | 61.9^h, i^ | 58.8^f^ | 49.7^i^ | 75.6^e^ |
| 26. | *Bacillus siamensis* NKIT26 | 82.9^c^ | 78.3^b^ | 65.0^d^ | 54.9^f, g^ | 71.9^g, h^ |
| 27. | *Bacillus safensis* NKIT27 | 75.7^e, f, g^ | 66.3^f, g^ | 58.7^f^ | 51.7^h, i^ | 70.1^i, j^ |
| 28. | *Bacillus siamensis* NKIT28 | 89.5^a^ | 78.5^b^ | 70.0^b^ | 69.8^c^ | 77.0^c, d, e^ |
| 29. | *Bacillus siamensis* NKIT29 | 76.7^d, e, f^ | 65.8^g^ | 67.1^c^ | 52.8^g, h^ | 75.9^d, e^ |
| 30. | *Bacillus safensis* NKIT30 | 73.2^g, h, i^ | 49.8^o^ | 29.8^m^ | 29.2^o, p^ | 77.6^c^ |
| 31. | *Bacillus safensis* NKIT31 | 53.7^r^ | 52.5^n^ | 16.9^p^ | 6.1^v^ | 68.8^j, k^ |
| 32. | *Bacillus nakamurai* NKIT32 | 64.6^l, m^ | 60.1^i, j^ | 54.3^g, h^ | 46.1^j^ | 73.1^f, g^ |
| 33. | *Bacillus amyloliquefaciens* NKIT33 | 71.5^i^ | 65.7^g^ | 65.0^d^ | 73.0^b^ | 80.0^b^ |
| 34. | *Bacillus safensis* NKIT34 | 42.5^u^ | 38.9^s^ | 29.1^m^ | 18.7^u, t^ | 65.9^n, o, p^ |
| 35. | *Bacillus safensis* NKIT35 | 68.5^j^ | 60.0^i, j^ | 13.5^q^ | 31.0^n, o^ | 66.7^m, n^ |
| 36. | *Bacillus australimaris* NKIT36 | 57.5^q^ | 47.1^p, q^ | 16.0^p^ | 30.2^n, o, p^ | 77.2^c, d, e^ |
| 37. | *Bacillus safensis* NKIT37 | 77.5^d, e^ | 65.9^g^ | 52.9^h^ | 55.2^f, g^ | 77.2^c, d^ |
| 38. | *Planococcus ruber* NKIT38 | 65.5^k, l, m^ | 54.2^n^ | 16.3^p^ | 0.0^w^ | 60.3^s^ |
| 39. | *Bacillus australimaris* NKIT39 | 67.2^j, k^ | 48.5^o, p^ | 41.0^k, l^ | 32.0^m, n^ | 76.0^c, d^ |
| 40. | *Bacillus subtilis* NKIT40 | 66.6^j, k, l^ | 65.4^g^ | 60.0^f^ | 68.3^c^ | 71.4^h, i^ |
| 41. | *Bacillus safensis* NKIT41 | 67.4^j, k^ | 58.3^j, k^ | 0.0^s^ | 0.8^w^ | 62.8^r^ |
| 42. | *Bacillus australimaris* NKIT42 | 53.0^r^ | 23.2^t^ | 21.8^o^ | 18.8^t, u^ | 64.5^p, q^ |
| 43. | *Bacillus safensis* NKIT43 | 73.1^g, h, i^ | 44.3^r^ | 6.2^r^ | 21.7^s^ | 73.7^f^ |
| 44. | *Bacillus zhangzhouensis* NKIT44 | 55.0^r^ | 36.8^s^ | 40.1^l^ | 35.6^l^ | 67.0^l, m, n^ |
| 45. | *Bacillus safensis* NKIT45 | 72.7^h, i^ | 54.9^l^ | 46.0^i^ | 40.0^k^ | 70.1^i, j^ |

**TABLE S3:** Inhibition percentage of isolated bacterial endophytes from V2 variety against plant pathogenic fungi

| S.No. | Bacterial endophytic strains (V2) | Percentage inhibition of fungal pathogens (%) | | | | |
| --- | --- | --- | --- | --- | --- | --- |
|  |  | ***Rhizoctonia Solani*** | ***Verticillium lateritium*** | ***Botrytis cinerea*** | ***Fusarium solani*** | ***Alternaria solani*** |
| 1. | *Bacillus safensis* NKIT46 | 66.1^e^ | 44.5^e,f,g^ | 30.5^d^ | 31.7^e^ | 69.1^b^ |
| 2. | *Bacillus zhangzhouensis* NKIT47 | 55.6^f^ | 29.7^j^ | 34.9^c^ | 18.9^h^ | 69.0^b^ |
| 3. | *Bacillus safensis* NKIT48 | 72.8^a,b,c^ | 28.5^j^ | 23.0^e^ | 33.6^d^ | 60.1^d^ |
| 4. | *Bacillus safensis* NKIT49 | 66.3^e^ | 33.1^i^ | 16.5^f^ | 31.6^e^ | 60.8^d^ |
| 5. | *Bacillus safensis* NKIT50 | 64.7^e^ | 44.3^e,f,g^ | 34.1^c^ | 28.2^f^ | 54.4^f^ |
| 6. | *Bacillus australimaris* NKIT51 | 64.8^e^ | 42.4^g^ | 22.3^c^ | 18.2^h,i^ | 65.0^c^ |
| 7. | *Bacillus australimaris* NKIT52 | 71.3^b,c,d^ | 21.4^k^ | 11.1^h,i^ | 3.2^m^ | 42.5^i^ |
| 8. | *Bacillus safensis* NKIT53 | 57.7^f^ | 33.9^i^ | 28.2^d^ | 22.5^g^ | 30.7^l^ |
| 9. | *Bacillus safensis* NKIT54 | 56.3^f^ | 11.5^l^ | 0.4^j^ | 1.2^o,p^ | 26.2^l^ |
| 10. | *Bacillus australimaris* NKIT55 | 65.0^e^ | 47.3^d,e^ | 13.7^g^ | 21.3^g^ | 33.2^k^ |
| 11. | *Bacillus zhangzhouensis* NKIT56 | 45.2^h^ | 21.2^k^ | 0.0^j^ | 0.0^p^ | 19.6^o^ |
| 12. | *Bacillus safensis* NKIT57 | 66.3^e^ | 44.7^e,f,g^ | 18.4^f^ | 10.9^k^ | 43.5^h,i^ |
| 13. | *Bacillus australimaris* NKIT58 | 65.5^e^ | 27.9^j^ | 9.6^h,i^ | 0.0^p^ | 42.7^i^ |
| 14. | *Bacillus australimaris* NKIT59 | 64.0^e^ | 32.6^i^ | 29.0^d^ | 0.0^p^ | 44.2^h,i^ |
| 15. | *Bacillus safensis* NKIT60 | 73.5^a,b,c^ | 33.8^i^ | 24.6^e^ | 12.7^j,k^ | 45.7^h,g^ |
| 16. | *Bacillus amyloliquefaciens* NKIT61 | 74.5^a^ | 63.6^a^ | 43.0^a^ | 57.4^a^ | 71.5^a^ |
| 17. | *Bacillus australimaris* NKIT62 | 63.6^e^ | 52.9^c^ | 24.9^e^ | 9.4^l^ | 43.8^h,i^ |
| 18. | *Bacillus zhangzhouensis* NKIT63 | 55.9^f^ | 54.2^c^ | 35.8^c^ | 34.1^d^ | 55.7^f^ |
| 19. | *Bacillus zhangzhouensis* NKIT64 | 45.6^g,h^ | 47.8^d^ | 23.5^e^ | 12.7^j,k^ | 44.1^h,i^ |
| 20. | *Bacillus safensis* NKIT65 | 78.7^a,b,c^ | 43.9^f,g^ | 28.7^d^ | 16.8^i^ | 39.1^j^ |
| 21. | *Bacillus safensis* NKIT66 | 73.6^a,b,c^ | 45.7^d,e,f^ | 43.1^a^ | 36.5^c^ | 58.5^d^ |
| 22. | *Bacillus zhangzhouensis* NKIT67 | 71.8^a,b,c^ | 42.9^f^ | 43.0^a^ | 39.9^b^ | 47.4^g^ |
| 23. | *Bacillus australimaris* NKIT68 | 74.1^a,b^ | 58.5^b^ | 43.8^a^ | 13.8^h,j^ | 14.5^p^ |
| 24. | *Bacillus australimaris* NKIT69 | 65.7^e^ | 36.8^h^ | 9.3^i^ | 3.0^n,o^ | 61.1^d^ |
| 25. | *Bacillus australimaris* NKIT70 | 66.0^e^ | 28.9^j^ | 0.0^j^ | 0.0^p^ | 58.1^e^ |
| 26. | *Bacillus zhangzhouensis* NKIT71 | 48.0^g^ | 23.0^k^ | 0.0^j^ | 0.0^p^ | 22.8^h,g^ |
| 27. | *Bacillus australimaris* NKIT72 | 69.0^d^ | 45.1^d,e,f,g^ | 38.8^b^ | 28.1^f^ | 47.5^g^ |
| 28. | *Bacillus australimaris* NKIT73 | 57.9^f^ | 45.7^d,e,f^ | 0.0^j^ | 0.0^p^ | 58.1^e^ |
| 29. | *Bacillus safensis* NKIT74 | 71.1^c,d^ | 52.7^c^ | 11.9^g,h^ | 5.0^m^ | 64.8^c^ |
| 30. | *Bacillus australimaris* NKIT75 | 63.8e | 32.7^i^ | 24.9^e^ | 18.2^h,i^ | 42.3^i^ |

**TABLE S4.** Main mass peaks of the lipopeptides produced by *Bacillus siamensis* strain NKIT9 mass spectrometry

| Mass peaks (m/z) | Assignment |
| --- | --- |
| 994.8 | C12 Surfactin[M + H^+^]^+^ |
| 1008.77 | C13 Surfactin[M + H^+^]^+^ |
| 1022.72 | C14 Surfactin [M + H^+^]^+^ |
| 1036.74 | C15 Surfactin [M + H^+^]^+^ |
| 1050.75 | C16 Surfactin [M + H^+^]^+^ |
| 1064.77 | C17 Surfactin [M + H^+^]^+^ |
| 1096.86 | Linear C18 Surfactin |
| 1045.77 | C15 Bacillomycin D [M + H^+^]^+^ |
